# Anti-Trypanosomal Activity of Nigerian Plants and Their Constituents

**DOI:** 10.3390/molecules20057750

**Published:** 2015-04-28

**Authors:** Ngozi Justina Nwodo, Akachukwu Ibezim, Fidele Ntie-Kang, Michael Umale Adikwu, Chika John Mbah

**Affiliations:** 1 Department of Pharmaceutical and Medicinal Chemistry, University of Nigeria, Nsukka 410001; Nigeria; E-Mails: saintakaben@yahoo.com (A.I.); cjmbah123@yahoo.com (C.J.M.); 2 Department of Chemistry, Chemical and Bioactivity Information Centre, Faculty of Science, University of Buea, P.O. Box 63, Buea 00237, Cameroon; E-Mail: ntiekfidele@gmail.com; 3 Department of Pharmaceutics, University of Nigeria, Nsukka 410001, Nigeria; E-Mail: adikwum@yahoo.com

**Keywords:** Nigeria, trypanosomiasis, medicinal plants, plant products

## Abstract

African trypanosomiasis is a vector-borne parasitic disease causing serious risks to the lives of about 60 million people and 48 million cattle globally. Nigerian medicinal plants are known to contain a large variety of chemical structures and some of the plant extracts have been screened for antitrypanosomal activity, in the search for potential new drugs against the illness. We surveyed the literatures on plants and plant-derived products with antitrypanosomal activity from Nigerian flora published from 1990 to 2014. About 90 plants were identified, with 54 compounds as potential active agents and presented by plant families in alphabetical order. This review indicates that the Nigerian flora may be suitable as a starting point in searching for new and more efficient trypanocidal molecules.

## 1. Introduction

African trypanosomiasis is a parasitic disease caused by a protozoan of the genus *Trypanosoma*. *Trypanosoma vivax* (*T. vivax*), *Trypanosoma congolense* (*T. congolense*) and to a lesser extent *Trypanosoma brucei brucei* (*T. b. brucei*) are the main species responsible for African animal trypanosomosis (AAT) called nagana in West Africa while *T. b. rhodesiense* and *T. b. gambiense* cause sleeping sickness (human African trypanosomiasis, HAT). Surra and Dourine are caused by the other trypanosome species *T. evansi* and *T. equiperdum* respectively. The disease is transmitted by a bite of the vector—tsetse fly (*Glossina* species) [1].

In Nigeria, trypanosomiasis seems to be re-emerging as an important livestock disease, assuming major clinical importance in small ruminants and extending to previously designated tsetse-free zones [2,3]. Apart from the old Gboko endemic focus remaining active, there have been reports of the disease outbreak in many other communities in Nigeria [4,5].The prevalence rate in different breed of animals in Nigeria for the past few years have been studied and ranged from 8.4% to 15.53% [6,7].

In Africa, the annual loss in livestock production and mixed agriculture alone due to the disease is valued at 5 billion US dollars. In 1995, WHO Expert Committee estimated that 60 million people were at risk with an estimated 300,000 new cases per year in Africa, with fewer than 30,000 cases diagnosed and treated. In 2004, the number of new reported cases fell to 17,616 and WHO considered in that due to increased control, estimated cumulative rate to be between 50,000 and 70,000 cases. In 2009, the number of new cases reported dropped below 10,000 (9878) for the first time in 50 years and the estimated number of actual cases is currently 30,000. This trend has been maintained in 2012, with 7216 cases reported [8,9,10].

The current chemotherapy of HAT relies on only six drugs (suramin, pentamidine, melarsoprol, eflorinithine, arsobal and mel B), five of which were developed more than 30 years ago. Others such as homidium, isometamidium and diminazene aceturate are used in animal infections. Each of these drugs has one or more of these challenges: expensive, highly toxic, need parenteral administration and parasites increasing resistance. However, tireless effort being made by WHO, private partners and local governments to eliminate HAT is yielding significant success. The Drugs for Neglected Diseases *initiative* (DND*i*) is developing fexinidazole to a new oral drug for HAT with a good chance of success. It has entered Phase II/III clinical study in patients with late-stage sleeping sickness. It is hoped that fexinidazole would solve the problems and limitations of current chemotherapeutic options [11,12,13].

Several reviews on medicinal plants used in treatment of trypanosomiasis have been published [14,15,16,17,18,19,20,21]. It is estimated that 66%–85% of the World’s population depends directly on plants as medicine and search for drugs derived from plants has accelerated in recent years [22,23,24,25,26,27,28,29,30]. Nigeria, located in West Africa on the Gulf of Guinea, has a rich biodiversity. There are many reports documenting the potentials of medicinal plants in Nigeria against several diseases except trypanosomiasis [31,32]. This paper documents works on Nigerian medicinal plants and derived products as source of trypanocidal agents which could be further investigated for the development of better drug molecules for the disease. We present the plants which are 84 by their plant families (40) in alphabetical order.

## 2. Acanthaceae, Amaryllidaceae, Anacardiaceae, Annonaceae, Apocynaceae, Araceae, Asclepiadaceae, Asteraceae and Burseraceae

**Figure 1:**
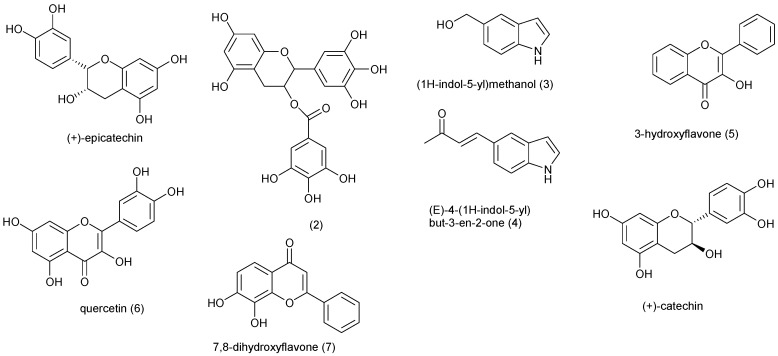
Chemical structures of compounds **1**–**8**.

Plants produce a great diversity of substances that could be active in many fields of medicine. Natural products from plant are proven template for new drug development [33]. The plants in these families are summarized in Table 1 and the compounds isolated from them are shown in Figure 1. *Peristrophe bicalyculata* (Acanthaceae) is found almost throughout India, Afghanistan and Africa. The herb is used against tuberculosis, snake poison, in bone fracture, sprain, fever, cold, and cough treatments [34]. 50 mg/kg of Cold water whole plant extract of *P. bicalyculata* immobilized 90% of *T. b. brucei in vitro* after one hour of incubation, while the methanol extract of the plant showed a dose-dependent suppressive property in mice infected with *T*. *evansi* [35,36]. Nok and Williams described that the extract obtained from *Allium sativum* (Amaryllidaceae) completely eliminated trypanosomes in mice on administering 120 mg/kg live weight at 4 days post-treatment. *A. sativum* is thought to have caused cell death in trypanosomes by inhibiting the synthesis of membrane lipids of the cell [37,38]. The aqueous methanol root extracts of *Lannea kerstingii* and *Mangifera indica* from Anacardiaceae and petroleum ether root extract of *Annona senegalensis* (Annonaceae) at 4 mg/mL, stopped motility of *T. brucei in vitro* within an hour of incubation [39]. In another study, Adeiza *et al.* tested *in vitro* trypanocidal activity of *A. senegalensis* and found that the crude extract immobilized *T. evansi* at 10 mg/mL [40,41]. A fraction obtained from an aqueous leaf extract of *Holarrhena africana* (Apocynaceae) completely cleared *T. b. rhodesiense* at a dose of 40 mg/kg bw i.p. in infected mice for 5 days post treatment [42]. Hexane and methanol extracts of *Spondias mombim* (Anacardiaceae) root yielded Compounds **1** and **2** respectively and compounds **3** and **4** were obtained from the ethyl acetate seed extract of *Monodora myristica* (Annonaceae). Compounds **1**, **3** and **4** were active *in vitro* against *T. b. brucei* with minimum inhibition concentration (MIC—μg/mL) of 25, 12.5 and 25 respectively [43]. The leaf extract of *Lannea welwistchii* showed trypanocidal activity (MIC = 6.3 mg/mL) against *T. b. brucei* [44]. *Haematostaphis barteri* is used by traditional medical practitioners in the north-eastern Nigeria to treat and manage trypanosomiasis [45]. Using short assay duration of 30 min, 0.5 mg/mL of *H. barteri* aqueous extract immobilize *T. b. brucei* and *T. congolense* [46].

*Carissa spinarum*, also known as the conkerberry or bush plum, is a large shrub that belongs to the Apocynaceae*.* Its ethanol root extract has been shown to have *in vivo* activity against *T. b. brucei* at ≥100 mg/kg body weight in infected mice [47]. Using a one hour exposure time, methanol extracts of *Adenium obesum* stem bark (Apocynaceae) and *Anchomanes difformis* rhizome (Araceae) stopped 50% of the motility of *T. b. brucei in vitro* at 4 mg/mL [39,48]. *In vivo* trypanocidal activity of *Carrisa edulis* (Apocynaceae) against *T*. *congolence* infection in rats was investigated using a methanol root extract. Oral treatment at different doses did not significantly clear the parasitemia, however, animals treated with 100 mg/kg/day survived longer than those treated with 200 mg/kg/day and the infected control group [49]. *Saba florida* (Apocynaceae) is traditionally eaten as an antidote against vomiting, diarrhoea and food poisoning [50]. *S. florida* aqueous methanol leaf extract (400 mg/kg) exhibited *in vivo* activity by clearing *T. b. brucei* in infected rats after 7 days [51]. *Gongronema latifolium* (Asclepiadaceae) has been reported to stop motility of *T. congolense* after about 10 min of *in vitro* treatment using 400 mg/kg of the whole plant’s methanol extract [52]. The ethyl acetate and methanol extracts of *Tridax procumbens* (Asteraceae), in contrast to extracts obtained with other solvents, were trypanocidal towards *T. b. brucei* at 200 mg/kg [53]. Further investigation of the ethyl acetate extract of *T. procumbens* led to the isolation of four flavonoids; 3-hydroxyflavone (**5**), quercetin (**6**), 7,8-dihydroxyflavone (**7**) and catechin (**8**). Compounds **6** and **7** were described to exhibit trypanocidal activity *in vitro* and *in vivo* as pure compounds without affecting normal human cell [54,55,56]. *In vitro* studies showed *T. b. brucei* was immobilized by 0.4 mg/mL of *Artemisia maritime* (Asteraceae) chloroform and petroleum ether extracts using short assay duration of less than one hour [57]. *Boswellia dalzielii* (Burseraceae), a tree of the Savannah forest of Nigeria, is used for the treatment of wound, diarrhoea, syphilis and to induce vomiting [58,59]. Freiburghaus *et al.* found that the trypanocidal activity of *B. dalzielii* varies according to extraction medium and part of plant used. Based on this, Atawodi *et al.* tested activities of extracts of different parts of *B. dalzielii* against *T. b. brucei*. His results revealed that methanol leaf, stem and root bark extracts of the plant at 10 mg/mL significantly immobilized the trypanosome [60,61].

**Table 1:** Plants from Nigeria with activity against African trypanosomes.

Family	Species	Traditional Uses	Plant Part	Ref.
Acanthaceae	*Peristrophe bicalyculata*	skin diseases, antidote for snake poison, diabetes	WP	[34]
Amaryllidaceae	*Allium sativum*	diabetes, tetanus, swellings	WP	[37]
Anacardiaceae	*Lannea kerstingii*	diarrhoea, cancer	R	[39]
	*Mangifera indica*	clearing digestion and acidity due to pitta	R	[39]
	*Spondias mombim*	used as febrifuge and diuretic	R	[43]
	*Lannea welwistchii*	diarrhoea, dysentery, dropsy	L	[44]
	*Haematostaphis barteri*	trypanosomiasis	SB	[45]
Annonaceae	*Monodora myristica*	stomachic, headaches, sores	SD	[43]
	*Anonna senegalensis*	food and pneumonia	L, R, SB	[40]
Apocynaceae	*Carissa spinarum*	analgesic	R	[47]
	*Adenium obesum*	arrow poison for hunting	R	[39]
	*Carrisa edulis*	rheumatism, stomach disorder	R, B, L	[49]
	*Holarrhena africana*	dysentery, diarrhoea, snakebite, infertility, malarial, diabetics	L	[42]
	*Saba florida*	rheumatism, antidote against vomiting, diarrhoea and food poison	L	[50]
Araceae	*Anchomanes difformis*	diabetes, diarrhoea	R	[48]
Asclepiadaceae	*Gongronema latifolium*	diabetes, high blood pressure	L, SB	[52]
Asteraceae	*Tridax procumbens*	inflammatory, microbial and protozoal diseases	WP	[53]
	*Artemisia maritima*	worm, stomachic infusion	WP	[57]
Burseraceae	*Boswellia dalzielii*	wound healing, diarrhea, syphilis, induce vomiting	L, SB, Re	[59]

R = root, RB = root bark, SB = stem bark, L = leaves, WP = whole plant, Re = rhizome, B = bark.

## 3. Capparaceae, Celastraceae, Clusiaceae, Combretaceae, Cucurbitaceae, Ebenaceae and Euphorbiaceae

**Figure 2:**
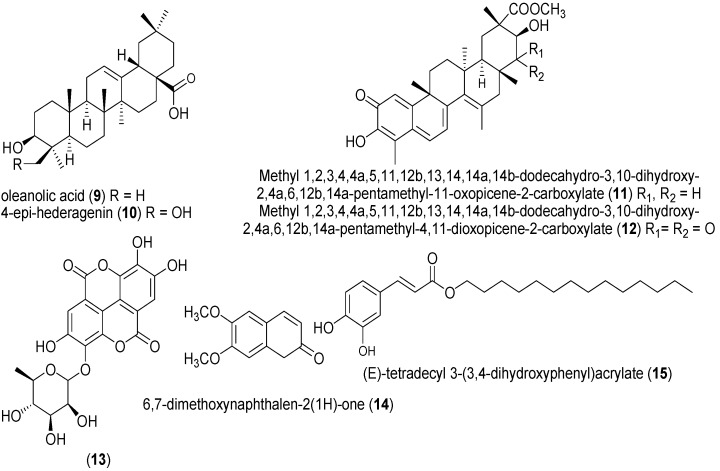
Chemical structures of compounds **9** to **15**.

The plants in the families listed above are summarized in Table 2 and potential active isolates are represented in Figure 2. Leaves of *Crateva adansonii* (Capparaceae) are used to treat ear infections while its root is employed to treat syphilis, jaundice and yellow fevers [62]. The ethyl acetate and hexane crude extract of the plant demonstrated moderate *in vitro* activity (MIC 12.5 μg/mL) against *T. b. brucei* [63]. Two phytoconstituents (oleanolic acid (**9**) and 4-epi-hederagenin (**10**)), which were not tested for activity, had also been isolated from *C. adansonii* and could account for the activity of the plant. The seed extract of *Bucholzia coriacea* (Capparaceae) is locally used in treatment of feverish conditions in Eastern Nigeria [64]. *T. b. brucei* was cleared in infected mice after administering 1000 mg/kg of aqueous and methanol seed extracts of *B. coriacea* i.p. for five consecutive days [65,66]. MIC of 0.625 μg/mL has been reported for both compounds **11** and **12** isolated from the hexane-ethyl acetate fraction of *Maytenus laevis* (Celastraceae) root [44,67]. The highest *in vivo* trypanocidal activity of *Garcinia kola* (Clusiaceae) seeds was observed in the alkaloid fraction which brought about 92.25% reduction in parasitaemia at 100 mg/kg in *T. b. brucei* infected rats. The antitrypanosomal property of the alkaloids from *G. kola* has been suggested to be due to DNA intercalation in combination with protein biosynthesis inhibition [68].

*Anogeissus leiocarpus* (Combretaceae) is a tree widely distributed in northern Nigeria. The aqueous methanol bark extract of *A*. *leiocarpus* had the highest *in vitro* antitrypanosomal activity out of all the other parts of the plant. 200 mg/kg of the extract made *T. b. brucei* immotile after 10 min incubation. Furthermore, the extract (200 mg/kg) was analyzed *in vivo* using *T. b. brucei* infected rats. Although it did not clear parasitemia in experimental rats after seven days, the rats survived longer than the infected control group [69]. The hexane-ethyl acetate extract of *Terminalia avicennioides* (Combretaceae) bark inhibited *T. b. brucei* activity *in vitro* with MIC of 2.5 μg/mL [43,70,71]. Compound **13** was isolated from *T*. *avicennioides* but did not show activity. Methanol extracts of *Terminalia superba* (Combretaceae) root and stem were effective with MIC value of 3.1 mg/mL each against *T. b. brucei in vitro* [44]. Using a short assay duration of about an hour revealed that 10 mg/mL of both *Momordica balsamina* (Cucurbitaceae) and *Diospyros Mespiliformis* (Ebenaceae) methanol extract drastically reduced motility of *T. b. brucei* [39,48]. Two plants of Euphorbiaceae family whose crude extracts showed antitrypanosomal activity, yielded two active compounds; compound **14** (*Euphorbia poisonii*) and compounds **15** (*Alchornea cordifolia*). Compounds **14** and **15** had activity with (MIC of 1.56 μg/mL and < 0.2 μg/mL respectively) against *T. b. brucei* [43].

**Table 2:** Plants from Nigeria with activity against African trypanosomes.

Family	Species	Traditional Uses	Plant Part	Ref.
Capparaceae	*Crateva adansonii*	stomach troubles, syphilis, jaundice and yellow fevers	L	[62]
	*Buchholzia coriacea*	feverish, malaria	SD	[64]
Celastraceae	*Maytenus laevis*	anti-inflammatory, analgesic	R	[67]
Clusiaceae	*Garcinia kola*	purgative, antiparasitic, antimicrobial	SD	[68]
Combretaceae	*Anogeissus leiocarpus*	trypanosomiasis, babesiosis	R, SB	[69]
	*Terminalia avicennioides*	cancer, fungal, bacterial infections	B	[70]
	*Terminalia superba*	furniture making and musical instrument	B	[44]
Cucurbitaceae	*Mormordica balsamina*	used to treat wound	WP	[48]
Ebenaceae	*Diospyros mespiliformis*	styptic to staunch bleeding, leprosy	L	[39]
Euphorbiaceae	*Euphorbia poisonii*	latex used as pesticides	B	[43]
	*Alchornea cordifolia*	eye treatment, venereal diseases	S	[43]

R = root, RB = root bark, SB = stem bark, L = leaves, S = stem, WP = whole plant, Re = rhizome, B = bark, SD = seed.

## 4. Fabaceae, Fagaceae, Hymenocardiaceae, Lamiaceae, Lauraceae, Loganiaceae, Lythraceae, Malvaceae and Melastomataceae

**Figure 3:**
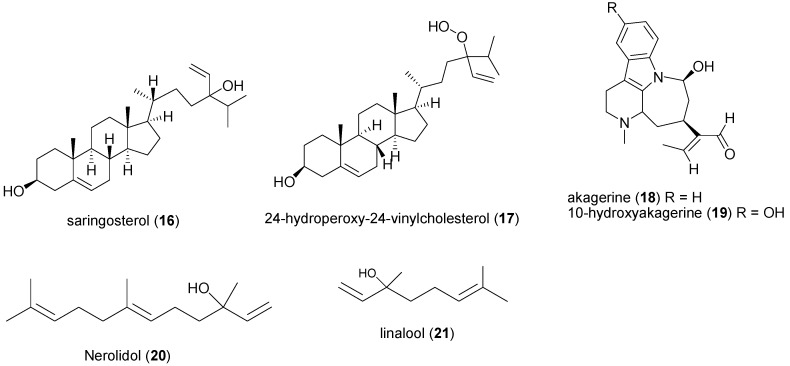
Chemical structures of compounds **16** to **21**.

The compounds isolated from plants of the families listed above are shown in Figure 3 and summarized in Table 3. Northern and South-Western Nigerians use *Acacia nilotica* (Fabaceae) to treat dysentery, tuberculosis and diabetes. Methanol extract (400 mg/kg) of *A. nilotica* stem bark cleared *T. b. brucei* in infected mice within eight days [72,73]. *In vitro* activities of petroleum ether extract of *Afzelia africana* leaves, aqueous extract of *Parkia clappertoniana* root, aqueous extractof *Piliostigma reticulatum* leaves, chloroform extract of *Prosopis africana* stem bark, methanol extract of *Afrormosia laxiflora* leaves, chloroform extract of *Erythrophleum suaveolus* stem bark, methanol extract of *Lonchocarpus laxiflorus* stem bark and chloroform extract of *Swartzia madagascariensis* root (all from the Fabaceae family) against *Trypanosoma* species have been described using short assay durations of less than an hour [39,48,74,75,76]. Antia *et al.* further demonstrated that methanol extract of *A. africana* leaves and stems were active against *T. b. brucei in vitro* with MLC (mg/mL) values of 3.1 and 12.5, respectively [44]. *Senna occidentalis* (Fabaceae) is a weed distributed throughout the tropical and subtropical regions of the World. It has been reported as a remedy for bacterial and malaria infections [77,78,79,80,81]. The ethanol extract of *S. occidentalis* leaf at 6.66 mg/mL concentration, eliminated *T. b. brucei in vivo* in 10 min in infected rats. Acute anaemia recorded in the *T. b. brucei* infected rat is a consistent feature of *Trypanosoma* infection and the treatment with the extract was able to significantly (*p* < 0.05) ameliorate the disease-induced anaemia. Methanol extract of *Quercus borealis* (Fagaceae) leaves exhibited a significant trypanocidal activity *in vitro* towards *T. evansi* by reducing the average mean trypanosomes counts from initial concentration (40.00 ± 0.00) at 250 μg/mL and completely killing of trypanosomes at 9 h of incubation at the same concentration [82]. Denise and Barret proposed that constituents of *Q. borealis* exhibited trypanocidal action by intercalation with DNA of the parasite, blockage of glycolysis pathway and interference with flagella [83].

In folkloric medicine of Idoma people of North Central Nigeria, *Hymenocardia acida* (Hymenocardiaceae) is used alone or in combination to treat trypanosomiasis and other fever related diseases [84]. Di-(2-ethylhexylphthalate (DEHP), friedelan-3-one, betulinic acid, lupeol, β-sitosterol, stigmasterol, oleic acid and homoorientin were isolated from the plant [85,86,87,88,89,90]. Aqueous ethanol extract of *H. acida* stem bark was active against *T. b. brucei in vitro* at MIC of 2.5 mg/mL in half an hour. At this concentration (2.5 mg/mL), morphology of red blood cells was unaffected [91]. None of the compounds from *H. acida* was tested for antitrypanosomal activity. The ethyl acetate extract of *Ocimum gratissimum* (Lamiaceae) leaves showed high antitrypanosomal *in vitro* activity (IC_50_ of 2.08 ± 0.01 μg/mL) and a high selective index of 29 [92]. *In vitro* analysis of chloroform extract of *Sterculia setigera* root revealed that 4 mg/mL of the extract only slightly reduced motility of *T. b. brucei* and *T. congolense* after 60 min exposure time [39]. Methanol extract of *Cassytha filiformis* (Lauraceae) stem (4 mg/mL) stopped *T. b. brucei* mobility after less than half an hour incubation time [48]. *T. b. brucei* (100%) was completely immobilized *in vitro* at MIC of 20 mg/mL of aqueous ethanol extract of *Anthocleista vogelii* (Loganiaceae) root bark [86,93,94,95].

In North-Eastern Nigeria, leaves and fruits of *Strychnos spinosa* (Loganiaceae) are consumed by lactating Fulani women to stimulate breast milk production [96]. Dichloromethane leaf extract of the plant yielded: saringosterol (**16**), 24-hydroperoxy-24-vinylcholesterol (**17**), akagerine (**18**) and 10-hydroxyakagerine (**19**). Out of the four compounds, only **16** and **17** exhibited significant antitrypanosomal *in vitro* activity with IC_50_ values of 7.8 ± 1.2 and 3.2 ± 1.2 µM respectively [97]. The essential oil from the plant’s leaves was active on *T. b. brucei* with IC_50_ 13.5 µg/mL. Hoet *et al.*, went further to show that nerolidol (**20**) and linalool (**21**), components of this oil, had a higher potency on the trypanosomes with IC_50_ values of 7.6 and 16.3 µM [98]. Ten and 4 mg/mL methanol extracts of *Tamianthus globiferus* (Loranthaceae) and *Lawsonia inermis* (Lythraceae) leaves respectively drastically ceased motility of *T. congolense* within 5 min [39,99]. A dose of 200 mg/kg of ethyl acetate extract of *Punica granatum* (Lythraceae) leaf reduced anaemia and promoted weight gain in mice infected with *T. b. brucei* [100]. The stem barks of *Bombax buonopozense* (Malvaceae) and *Heterotis rotundifolia* (Melastomataceae) methanol extracts (200 and 300 mg/kg body weight, respectively) cleared *T. b. brucei* within seven days of treatment duration, while 67% of *T. b. brucei* infected mice survived for over 43 days when treated with 400 mg/kg methanol seed extract of *Adansonia digitata* (Malvaceae) [70,101,102]. *Dissotis rotundifolia* (Melastomataceae) is a medicinal plant widely used in Nupe (Nigeria) ethno-medicine to treat trypanosomiasis [103,104]. Oral and i.p. administration of 800 mg/kg of ethanol leaf extract of the plant to rat infected with *T. b. brucei* significantly reduced parasitemia by 66.7% and 78.4%, respectively. The parasite was killed within 45 s of *in vitro* exposure to the same concentration of the extract. *Hyptis spicigera* (Lamiaceae) is locally known as “Bunsuru fadama” in Hausa language, Northern Nigeria. When the plant is crushed and applied to the head, it relieves headache [59]. Findings by Ladan *et al.*, demonstrated that 0.5 µg/mL of the volatile oil from *H*. *spicigera* leaves, killed *T. b. brucei in vitro* within 6 min of administration. GC-MS was used to analyze the chemical composition of the essential oil and about 30 constituents were identified [105,106,107,108].

## 5. Meliaceae, Moraceae, Moringaceae, Myrtaceae, Ochnaceae, Phyllanthaceae, Poaceae, Polygalaceae, Rubiaceae, Rutaceae, Solanaceae, Ulmaceae, Verbenaceace, Vitaceae, Zingiberaceae

**Figure 4:**
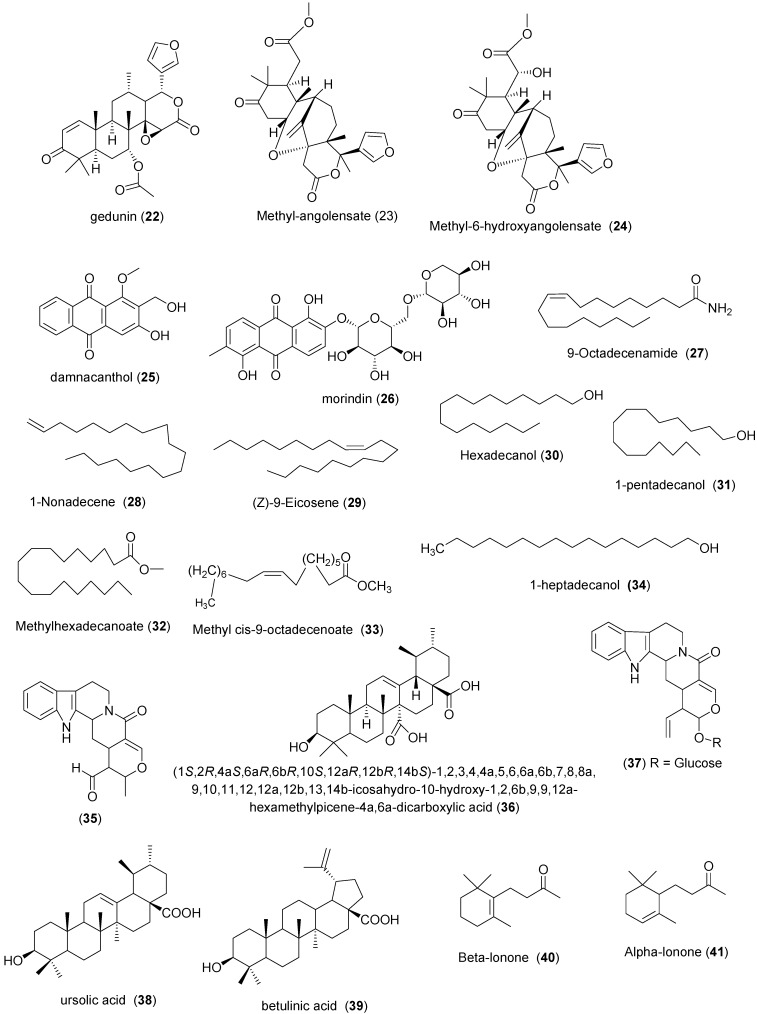

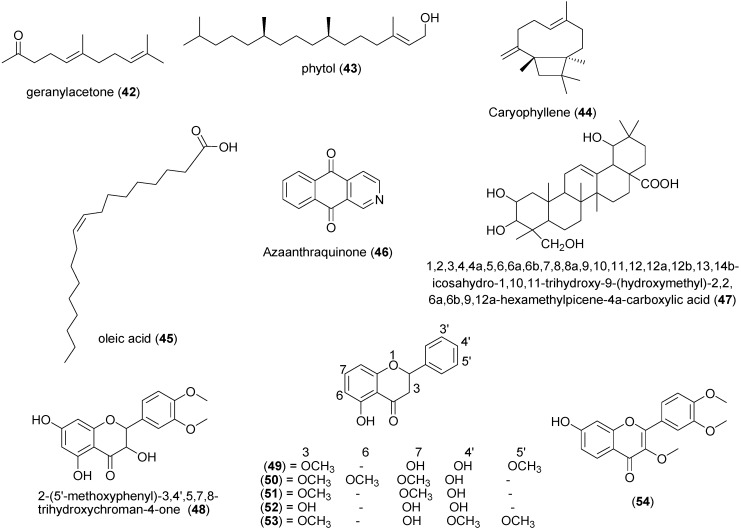
Chemical structures of compounds **22**–**54**.

A summary of the plants and derived products of the above listed families are in Table 4 and Figure 4 respectively. Various extracts of leaf, bark, stem and seed of *Khaya senegalensis* (Meliaceae) were reported to treat several human diseases [109]. Rats infected with *T. b. brucei* showed a significant decrease in blood parasite burden within six days when treated with aqueous stem bark extract of *K. senegalensis* at 60–100 mg·kg^−1^ bw i.p. [110]. Umar *et al.* reported that the stem bark of *K. sengalensis* possessed the highest *in vitro* activity among the six extracts tested as it eliminated the parasites within 5 min of incubation time at 1 mg/mL [111]. The following triterpenoids: gedunin (**22**); methyl-angolensate (**23**); methyl-6-hydroxyangolensate (**24**), isolated from *K. senegalensis* stem bark, though not tested, could be responsible for its trypanocidal activity [112]. aqueous stem bark extract of *Securidaca longepedunculata* (Polygalaceae) and methanol stem bark extract of *Pseudocedrella kotschi* (Meliaceae), methanol stem bark extract of *Ficus sycomorus* (Moraceae), chloroform stem bark extract of *Canarium schweinfurthii* (Poaceae) and chloroform stem bark extract of *Syzygium guineense* (Myrtaceae) (4 mg/mL) ceased *T. b. brucei* motility *in vitro* within the incubation time of less than one hour [39,113].

**Table 3:** Plants from Nigeria with activity against African trypanosomes.

Family	Species	Traditional Uses	Plant Part	Ref.
Fabaceae	*Acacia nilotica*	used to treat cancers and/or tumours of ear, eye	SB	[72,73]
	*Afzelia Africana*	trypanosomiasis, convulsion, hernia	WP	[74]
	*Parkia clappertoniana*	dental caries, conjunctivitis	R	[75]
	*Piliostigma reticulatum*	ulcer, boils, wounds, cancer, syphilis and diarrhoea	L	[76]
	*Prosopis Africana*	used to prepare food in Northern Nigeria	SB	[77]
	*Afrormosia laxiflora*	epilepsy and psychosis	L	[39]
	*Erythrophleum suaveolus*	arthritis, rheumatism, dropsy, swelling, eye treatment, laxative	SB	[39]
	*Lonchocarpus laxiflorus*	dermatitis, headache, intestinal worm, jaundice, ulcer, anthelmintic	SB	[48]
	*Swartzia madagascariensis*	poison arrow and fishing, insecticide	R	[48]
	*Senna occidentalis*	bacterial and malaria infections	L	[ 89]
Fagaceae	*Quercus borealis*	dyspnea, nausea, emesis, diarrhoea and muscular pain	L	[78]
Hymenocardiaceae	*Hymenocardia acida*	Hypertension	R, SB	[80]
Lamiaceae	*Ocimum gratissimum*	the oil is medicine for respiratory tract infections, diarrhoea, eye problem, skin diseases	L	[88]
	*Hyptis spicigera*	Cold, insecticides	L	[59]
Lauraceae	*Cassytha filiformis*	food and infectious diseases	L, S	[48]
Loganiaceae	*Anthocleista vogelii*	purgative, diuretic, ulcer, stomach-ache	R, SB	[87]
	*Strychnos spinosa*	taken by lactating women to stimulate breast milk production	L	[96]
Loranthaceae	*Tapinanthus globiferus*	hypertension epilepsy, relief pain, tinnitus and trypanosomiasis	L, SB	[99]
Lythraceae	*Lawsonia inermis*	used to adorn women’s bodies as part of social and holiday celebration	L	[39]
	*Punica granatum*	diarrhoea, dysentery	L	[100]
Malvaceae	*Bombax buonopozense*	to treat edema	SB	[102]
	*Adansonia digitata*	to treat cancer	SD	[101]
	*Sterculia setigera*	used as a thickener and emulsifier	R	[39]
Melastomataceae	*Heterotis rotundifolia*	malaria, rheumatism, diarrhoea	WP	[102]
	*Dissotis rotundifolia*	trypanosomiasis treatment	L	[104]

R = root, RB = root bark, SB = stem bark, L = leaves, S = stem, WP = whole plant, Re = rhizome, B = bark, SD = seed.

*Morinda lucida* (Rubiaceae) root together with *Mangifera indica*, *Carica papya* and *Cassia podocarba* leaves are used in Nigeria to treat malaria [114]. Adewunmi and Adesogan isolated some anthraquinones such as damnacanthol (**25**) and morindin (**26**) from *M. lucida*. Intraperitoneal administration of methanol leaf extract of *M. lucida* caused a significant reduction in parasitemia in *T. b. brucei* infested rats and mice [115,116]. An *in vitro* assay revealed that the petroleum ether extract of the root bark, chloroform extract of the stem bark, methanol extract of the stem and the aqueous extract of all parts of *Moringa oleifera* (Moringaceae) were active at 4 and 2 mg/mL doses against *T. b. brucei* [117]. The anti-trypanosomal screening of *Psidium guajava* (Myrtaceae) leaves revealed that it inhibited growth of *T. b. brucei* (IC_50_ of 6.3 μg/mL and 48.9 μg/mL) for 80% and 20% ethanol preparations respectively [118,119,120]. *Eucalyptus camaldulensis* (Myrtaceae) methanol leaf extract at a dose of 150 mg/kg body weight/day extended the lifespan of *T. b. brucei* infected mice by six days [120]. GC-MS analysis of *E. camaldulensis* fraction yielded 9-octadecenamide (**27**), 1-nonadecene (**28**), (*Z*)-9-eicosene (**29**), hexadecanol (**30**), 1-pentadecanol (**31**) for fraction A; and methyl hexadecanoate (**32**), methyl *cis*-9-octadecenoate (**33**), and 1-heptadecanol (**34**) for fraction B [121,122]. Aqueous extract *Lophira lanceolata* (Ochnaceae) Leaf and ethanol extract of *Gardenia erubescens* (Rubiaceae) stem had 100% activity *in vitro* against *T. b. brucei* and *T. congolense* at 20 mg/mL [92,123]. Peter *et al.* showed that 1000 µg/mL methanol extract of *Picrorhiza kurroa* (Plantaginaceae) rhizome completely killed *T. evansi in vitro* after an incubation time of 8 h [124].

Among the three compounds **35**, **36** and **37** isolated from the ethanol bark extracts of two plants from the Rubiaceae family (*Nauclea pobeguinii* and *Nauclea latifolia*), only **37** had activity *in vitro* (MIC = 12.5 μg/mL) against *T. b. brucei* [43,125,126]. Ten compounds with *in vitro* anti-trypanosomal properties; ursolic acid (**38**), oleanolic acid (**9**), betulinic acid (**39**), β-lonone (**40**), α-lonone (**41**), geranylacetone (**42**), phytol (**43**), caryophyllene (**44**) and oleic acid (**45**) have been isolated from *Keetia leucantha* (Rubiaceae). Compounds **38** and **9** demonstrated activities with IC_50_ of 2.5 and 7.3 μg/mL respectively towards *T. b. brucei* [127]. *Mitracarpus scaber* (Rubiaceae) is used in Nigeria to treat headache, toothache, venereal diseases, amenorrhoea, dyspepsia and leprosy [128]. Its methanol fraction yielded azaanthraquinone (**46**) which caused complete disappearance of *T. congolense* (*in vivo*) in mice at 50 mg/kg bw for 5 day without relapse. The authors suggested that the mechanism by which the compound performed its trypanocidal effect was by interfering with the mitochondrial electron transport system of the parasite [129]. *Ximenia americana* (Ochnaceae) is a plant used in traditional medicine for the treatment of malaria, ulcers, and infectious diseases. Aqueous extract of *X. americana* stem bark exhibited *in vitro* trypanocidal effect by immobilizing 90% of the *T*. *congolense*after 30 min incubation [123]. *Zanthoxylum zanthoxyloides* (Rutaceae) showed only trypanostatic effects and could not completely clear the *T. b. brucei in vivo* in infected mice [70].

Hexane whole plant extract of *Withania somnifera* (Solanaceae) had *in vitro* activity against *T. b. brucei* (MIC = 50 μg/mL) while 20 µg/mL of compound **47** isolated from the plant immobilized 78% of *T. b. brucei* [43]. Methanol leaves extracts of *Trema orientalis* (Ulmaceae) and *Vitex doniana* (Verbenaceace) exhibited *in vitro* activities with IC_50_ values of 3.50 and 6.58 μg/mL respectively against *T. b. rhodesiense* in less than one hour [70]. Aqueous methanol leaf extract of *Cissus multistriata* (Vitaceae) was very active against *T. b. brucei*. Parasitemia level in infected albino rats disappeared on sixth day of treatment with 400 mg/kg b.w of the plant extract intraperitoneally. Omale and Joseph suggested that the high trypanocidal activity of the plant could be due to its flavonoid content [51,130]. After 9 h of exposing *T. evansi*, *in vitro*, at 1000 μg/mL of methanol extract of *Zingiber officinale* (Zingiberaceae) rhizome, 95.86% of the parasites died [83]. Nwodo *et al.* reported that 2-(5'-methoxyphenyl)-3,4',5,7,8-trihydroxychroman-4-one (**48**), 2-(5'-methoxyphenyl)-4',5,7-trihydroxy-3-methoxychromen-4-one (**49**), penduletin (**50**), 2-(4'-hydroxy-phenyl)-5-hydroxy-3,7-dimethoxychromen-4-one (**51**), 2-(4-hydroxyphenyl)-3,5,7-trihydroxy-chromen-4-one (**52**), artemetin (**53**) and 2-(3',4'-dimethoxyphenyl)-7-hydroxychromen-4-one (**54**) from *Vitex simplicifolia* (Verbenaceace) leaf were active (IC_50_ values range 4.7–23.7 µM) against *T. b. rhodesiense* after less than one hour of incubation time. Compound **54** showed the most promising and selective trypanocidal activity (IC_50_ = 4.7 µM) with a selectivity index of 9.8. The authors observed that trypanocidal activity of the compounds increases with increase in methylation of hydroxyl groups. This is expected because methylation increases lipophilicity which increases permeability of molecule across membranes of the parasite [131,132,133].

**Table 4:** Plants from Nigeria with activity against African trypanosomes.

Family	Species	Traditional Uses	Plant Part	Ref.
Meliaceae	*Khaya senegalensis*	to treat malaria	L, AS	[109]
	*Pseudocedrella kotschi*	to treat diarrhoea, dysentery, epilepsy	SB	[39]
Moraceae	*Ficus sycomorus*	to treat snakebite, jaundice, dysentery	SB	[39]
Moringaceae	*Moringa oleifera*	to treat diabetes and intestinal worms	L, S, SB, R	[116]
Myrtaceae	*Psidium guajava*	to treat diarrhoea, hypertension	L	[118]
	*Syzygium guineense*	used to bath ill person	SB	[113]
	*Eucalyptus camaldulensis*	used to treat malaria and typhoid fevers	L	[120]
Ochnaceae	*Lophira lanceolata*	dermatosis, toothache, muscular tiredness	L, SB	[87]
	*Ximenia americana*	treatment of fever, jaundice, impotence, sleeping sickness	SB	[123]
Plantaginaceae	*Picrorhiza kurroa*	to treat asthma, bronchitis, chronic dysentery, malaria	WP	[124]
Poaceae	*Canarium schweinfurthii*	burnt for fumigation	SB	[39]
Polygalaceae	*Securidaca longepedunculata*	to treat cough, chest pain, toothache and diabetes	R	[113]
Rubiaceae	*Gardenia erubescens*	used as dye	L	[92]
	*Nauclea latifolia*	to treat fever, dental caries, sceptic mouth, malaria	B	[125]
	*Nauclea pobeguinii*	to treat fever, dental caries, sceptic mouth, malaria	B	[43]
	*Keetia leucantha*	to treat malaria	L	[127]
	*Mitracarpus scaber*	to treat headache, toothache, venereal disease, amenorrhoea, dyspepsia, leprosy	L	[128]
	*Morinda lucida*	used to treat malaria	L	[115]
Rutaceae	*Zanthoxylum zanthoxyloides*	stomach disorder, worm infection	SB	[70]
Solanaceae	*Withania somnifera*	to treat external tumors, tubercular glands and ulcer	WP	[43]
Ulmaceae	*Trema orientalis*	to treat cough, sore throats, asthma, bronchitis, gonorrhea, yellow fever, toothaches	L	[70]
Verbenaceace	*Vitex doniana*	anemia, gonorrhea, dysentery and to improve fertility	L	[70]
	*Vitex simplicifolia*	to treat malaria	L	[131]
Vitaceae	*Cissus multistriata*	for the management of protein deficiency	L	[130]
Zingiberaceae	*Zingiber officinale*	gastrointestinal diseases, dyspnea, nausea, emesis, diarrhoea and muscular pain	R	[83]

R = root, RB = root bark, SB = stem bark, L = leaves, S = stem, WP = whole plant, Re = rhizome, B = bark, SD = seed, AS = axial stem.

## 6. Conclusions

This review, the first of its kind on antitrypanosomal medicinal plants from the Nigerian flora, represents an overview of the potentials of these plants in combating the disease. It is intended to serve as the scientific baseline information for the use of documented plants as well as a starting point for future studies for the discovery of better trypanocidal molecule(s). Most of the plants were evaluated as crude extracts. Only compounds **1**, **3**, **4**, **9**, **11**–**12**, **14**–**17**, **20**–**21**, **37**–**38**, **47**–**54** were actually tested for antitrypanosomal properties. Mode of action of the compounds has almost never been thoroughly studied; only possible mechanisms have been suggested. This calls for more detailed investigations in this direction. In general, even though not all plants reviewed here are native or unique to Nigeria or West Africa, this survey suggests that Nigerian flora is a potential suitable starting point to discovering new and better trypanocidal drug molecules.
